# Structural and functional characterization of a hypothetical protein in the RD7 region in clinical isolates of *Mycobacterium tuberculosis* — an in silico approach to candidate vaccines

**DOI:** 10.1186/s43141-022-00340-5

**Published:** 2022-04-08

**Authors:** Kaviya Parambath Kootery, Suma Sarojini

**Affiliations:** Department of Lifesciences, CHRIST (Deemed to be University), Bengaluru, Karnataka 560029 India

**Keywords:** *Mycobacterium tuberculosis*, *M. canetti*, RD7, PSPIRED, Robetta, Immunogenic, Ramachandran plot, Flavodoxin-like fold, Membrane protein, Vaccine

## Abstract

**Background:**

*Mycobacterium tuberculosis* has been ravaging humans by inflicting respiratory tuberculosis since centuries. Bacillus Calmette Guerine (BCG) is the only vaccine available for tuberculosis, and it is known to be poorly effective against adult tuberculosis. Proteins belonging to the ESAT-6 family and PE/PPE family show immune responses and are included in different vaccine trials. Herein, we study the functional and structural characterization of a 248 amino acid long putative protein novel hypothetical protein 1 (NHP1) present in the RD7 region of *Mycobacterium tuberculosis* (identified first by subtractive hybridization in the clinical isolate RGTB123) using bioinformatics tools.

**Results:**

Physicochemical properties were studied using Expasy ProtParam and SMS software. We predicted different B-cell and T-cell epitopes by using the immune epitope database (IEDB) and also tested antigenicity, immunogenicity, and allergenicity. Secondary structure of the protein predicted 30% alpha helices, 20% beta strands, and 48% random coils. Tertiary structure of the protein was predicted using the Robetta server using the *Mycobacterium smegmatis* protein as the putative protein with homology. Structural evaluations were done with Ramachandran plot analysis, ProSA-web, and VERIFY3D, and with GalaxyWEB server, a more stable structure was validated with good stereo chemical properties.

**Conclusion:**

The present study of a subtracted genomic locus using various bioinformatics tools indicated good immunological properties of the putative mycobacterial protein, NHP1. Evidence obtained from the analyses of NHP1 using structure prediction tools strongly point to the fact that NHP1 is an ancient protein having flavodoxin folding structure with ATP binding sites. Positive scores were obtained for antigenicity, immunogenicity, and virulence too, implying the possibility of NHP1 to be a potential vaccine candidate. Such computational studies might give clues for developing newer vaccines for tuberculosis, which is the need of the hour.

**Supplementary Information:**

The online version contains supplementary material available at 10.1186/s43141-022-00340-5.

## Background

The World Tuberculosis Report, 2020, estimated ten million newly infected tuberculosis (TB) cases in 2019 of which 1.2 million people were HIV-negative and 2,08,000 people were HIV positive [1]. The only vaccine developed against TB is BCG, which is not very effective against adult tuberculosis [2]. Though many vaccines are in trial stages, significant efficacy has not yet been reported. Even MVA85A, one of the most promising among candidate vaccines, failed to give significant protection against the disease in a trial conducted on HIV patients [3]. Furthermore, *Mycobacterium tuberculosis* shows resistance to commonly used drugs, which makes the disease more difficult to treat [4]. The first whole genome sequencing of *M. tuberculosis* was done in 1998, which showed that more than half of the genome encodes hypothetical proteins [5]. Members belonging to the *M. tuberculosis* complex share 99% of genome sequence similarity but exhibit different pathogenic outcomes due to the coevolution of the species with animals and humans [6]. A large number of virulent mycobacterial genes studied were membrane-anchored genes associated with lipid metabolism [7]. Biological functions of hypothetical proteins with high GC content are predicted to play a major role in the pathology and virulence of the bacteria [8]. Comparative study of common genes in *M. tuberculosis* and *M. leprae* showed 219 genes unique for *Mycobacterium* species of which a few codes for highly conserved ESAT-6 family protein [9]. Proteins with low molecular weight encoding the ESAT-6 gene are widely used as diagnostic tools and in vaccine studies as they possess multiple epitopes for both B and T cells [10]. RD1, RD7, RD9, and RD11 regions of *M. tuberculosis* are considered as immunodominant regions. Genes specific to human isolates were identified in the RD7 region of H37Rv strain, for example, the *mce*3 gene (mammalian cell entry) located on the cell surface which plays an important role in the survival of the pathogen in the host [11]. RD7 regions were shown to possess eight mce-associated membrane proteins (Rv1966-Rv1973), two integral proteins (Rv1964, Rv1965), and one membrane protein (Rv1974). Although the function of mce genes is not completely studied, computational studies of mce2 genes have shown T-cell epitopes inducing property revealing the immunogenic nature of mce genes [12].

Computer-aided drug discovery studies in the field of tuberculosis research are a good model to find new drug targets against *M. tuberculosis* [13]. SMRT sequencing of the MTBC genome studies has shown that the genomes are highly conserved with 99% identity. Fifty-one secretory proteins of *M. tuberculosis* were identified using computational tools of which only seven secretory proteins were previously reported [14]. Hypothetical proteins belonging to different classes like enzymes, transporters, receptors, and structural proteins were studied using bioinformatics tools. Structure prediction, mutational analysis, and functional studies of different *M. tuberculosis* proteins were done using in silico methods of which few were predicted to have vaccine potential, ribosome binding regions, GTP binding domains, etc. [15]. Reverse vaccinology studies were done to develop highly immunogenic novel multiepitope sub-unit vaccines against tuberculosis [16].

Our present focus, the NHP1 locus, was obtained by subtractive hybridization in an earlier study using *M. tuberculosis* H37Rv and Indian clinical isolates. Using the initial 384 bp subtracted product as a probe for RFLP with different restriction enzymes followed by sequencing and assembly of the subcloned fragments, a 4.5 kb subtractive fragment was obtained by genome walking. Three potential ORFs were found in the 4.5 loci, NHP1 being one among them [17]. NCBI BLAST studies showed 99% sequence similarity with *Mycobacterium canetti* and predicted 5 potential ORFs. The present study is an in silico approach of the functional and structural studies of one of the novel hypothetical proteins (NHP1), present in the N4.5 locus of clinical isolates. Being a primitive protein and also being located in the RD7 region make NHP1 an ideal vaccine candidate to be explored. In order to check the probable use of NHP1 protein as a potential vaccine candidate against tuberculosis, various computational tools including Expasy ProtParam, SMS suite, NCBI CDD blast, PFAM, SMART, TB Pred, TMHMM, InterPro, VaxiJen, AllerTop, AllergenFP, VirulentPred, GOR4, SOPMA, PSIPRED, Robetta, PROCHECK, and GalaxyWEB server were used. Three positive and negative controls each were used for the comparison of results of the in silico study. The 3D structural studies of NHP1 were done using homology protein modeling. Proteins with high sequence similarity were selected as templates using the MODELLER server. Along with the homology template model, a position-specific scoring matrix (PSSM) and hidden Markov model (HMM)-based server Robetta were considered to develop the 3D structure of the query protein. The developed structures were refined and validated using different quality assessment tools including PROCKECK. The refinement of the developed 3D structure of the protein was also done using the GalaxyWEB server which is based on molecular dynamic simulation. The present study aimed at exploring the possibility of NHP1 protein as a potential vaccine candidate against tuberculosis, a disease for which there are no many vaccines in the market.

## Methods

### Sequence retrieval

The nucleotide sequence of N4.5 deposited earlier by our research group was retrieved from the National Center for Biotechnology Information (NCBI) GenBank (accession number — GU994138.2). NHP1 locus had 747 bp encoding a protein of 248 amino acids. The nucleotides encoding esxA (Rv3875, gene ID: 886209), esxB (Rv3874, gene ID: 886194), and fbpB (Rv1886c, gene ID: 885785) used as positive controls were retrieved from NCBI GenBank. whiB2 (Rv3260c, ID: 887598), tuf elongation factor (Rv0685, gene ID: 888262), and cyp144 (Rv1777, gene ID: 885839) used as negative control were also retrieved from NCBI GenBank. The physical and chemical characters, immunological properties, and the secondary and tertiary structures of the protein were studied using different bioinformatics tools.

### Physicochemical characterization

Expasy’s ProtParam [18] https://web.expasy.org/protparam/ server was used for physical and chemical characterization of the proteins. The prediction included molecular weight, isoelectric point, extinction coefficient, and instability index. SMS (Sequence Manipulation Suite) v2.0 [19] https://www.bioinformatics.org/sms2/ was also used to predict the physical properties using the sequence of 248 amino acids along with the positive and negative controls as input.

### Conserved domain search

Bioinformatics tools including CDD-BLAST [20] https://www.ncbi.nlm.nih.gov/Structure/cdd/ wrpsb.cgi, PFAM [21] http://pfam.xfam.org/ and SMART [22] http://smart.embl-heidelberg.de/smart/set_mode.cgi were used to search the conserved domains in the target protein and control samples.

### Subcellular localization

Subcellular localization prediction tools like TBpred [23] (https://webs.iiitd.edu.in/tbpred/submission.html), CELLO (sub**CEL**lular **LO**calization prediction) [24] (http://cello.life.nctu.edu.tw/), TMHMM [25] (http://www.cbs.dtu.dk/services/TMHMM/), SignalP (https://services.healthtech.dtu.dk/service.php?SignalP-5.0) and SOSUI [26] (https://harrier.nagahama-i-bio.ac.jp/sosui/) were used to study whether the protein is localized in the cytoplasm or on the cell surface. InterPro [27] (https://www.ebi.ac.uk/interpro/) tool which predicts functional analysis of proteins by comparing the referral sequences with predicted databases provided by different softwares was used to study the protein. Proteins are further classified into different families after domain prediction and site analysis. Furthermore, PFP-FunDSeqE (predicting protein fold pattern with functional domain and sequential evolution information) [28] (http://www.csbio.sjtu.edu.cn/bioinf/PFP-FunDSeqE/) server was used to find protein folding patterns based on functional domain and its evolutionary information. NHP1 protein along with positive and negative controls was studied using these servers.

### Prediction of antigenicity, allergenicity, and toxicity

VaxiJen v2.0 [29] (http://www.ddg-pharmfac.net/vaxijen/VaxiJen/VaxiJen.html) server was used to predict the antigenicity of the query proteins. Antigenicity of the protein was studied using the server ANTIGENpro (http://scratch.proteomics.ics.uci.edu/explanation.html). Allergenicity is very crucial in the development of vaccines. AllerTOP v.2.0 [30] (https://www.ddg-pharmfac.net/AllerTOP/) and AllergenFP [31] (http://ddg-pharmfac.net/AllergenFP/data.html) servers were used to predict the allergenicity of the protein. ToxinPred [32] (https://webs.iiitd.edu.in/raghava/toxinpred/algo.php) is a free server which helps in predicting the toxic regions of a protein sequence if any. This server can also be used to identify a new toxic peptide present in organisms.

### Virulence factor analysis

VirulentPred [33] (http://203.92.44.117/virulent/) was used to predict the virulence factors. VirulentPred is based on the SVM method with 81.8% accuracy.

### T-cell epitope prediction

HLAPred (http://crdd.osdd.net/raghava/hlapred/) and NetCTL [34] (http://www.cbs.dtu.dk/services/NetCTL/) servers were used to predict the T-cell epitopes of the proteins. The NetCTL server was used to predict cytotoxic T-lymphocyte (CTL) epitopes of the query proteins. NetCTL prediction depends on MHCI binding affinity, C-terminal cleavage function, and transporter function associated with antigen processing. IEDB MHCII [35] (http://tools.iedb.org/mhcii/) server was used for identifying helper T-lymphocyte (HTL) epitopes. Human/HLA-DR were chosen as the species/locus with 7 allele human leukocyte antigen (HLA), and 15-m-long epitopes were retrieved. The percentile rank was compared with the Swiss-Prot database showing high MHCII affinity. T-cell epitopes immunogenicity prediction tool was used to study if the predicted T-cell epitopes elicit an immune response. Immunogenicity of class-I peptide MHC complex (pMHC) (http://tools.iedb.org/immuno genicity/) is based on the positions and the properties of the amino acids.

### B-cell epitope prediction

BcePred [36] (https://webs.iiitd.edu.in/raghava/bcepred/bcepred_submission.html) and ABCpred [37] (https://webs.iiitd.edu.in/raghava/abcpred/ABC_method.html) were used for the prediction of linear B-cell epitopes. BepiPred by IEDB [38] (http://tools.iedb.org/bcell/) resources was also used to predict linear B epitopes from the protein structure. In this server, amino acid characteristic scales and hidden Markov Model were used to predict linear B-cell epitopes.

### Secondary structure prediction

Online PSI-blast-based secondary structure prediction (PSIPRED) [39] (http://bioinf.cs.ucl.ac.uk/psipred/) and self-optimized prediction method with alignment (SOPMA) [40] (https://npsa-prabi.ibcp.fr/NPSA/npsa_sopma.html) and GOR IV [41] (https://npsa-prabi.ibcp.fr/cgi-bin/npsa_automat.pl?page=npsa_gor4.html) servers were used to predict the secondary structure of NHP1 protein.

### Tertiary structure model

The protein tertiary structure was modelled using MODELLER [42] (https://salilab.org/modeller/) and Robetta [43] (https://robetta.bakerlab.org/) servers. The model protein structures obtained were validated using PROCHECK [44] (http://www.csb.yale.edu/Excite/AT-CSBquery.html) and VERIFY3D [45] (https://saves.mbi.ucla.edu/). PROCHECK was used to check the stereochemical parameters along with the bond angles and bond lengths of the protein structure. ProSA-web [46] (https://prosa.services.came.sbg.ac.at/prosa.php) server was used for Z-score validation. Galaxy Refine — GalaxyWEB server [47] (http://galaxy.seoklab.org/cgi-bin/help.cgi?key=METHOD&type= REFINE) was used to refine the predicted homology model.

## Results

### Physicochemical characterization

In silico studies of the putative 747 bp long gene in the N4.5 region of *M. tuberculosis* has revealed a 248 amino acid long protein — NHP1 — with a molecular weight of 26.34 kDa, pH 4.90 (highly acidic), with a theoretical pI 5.09, and estimated half-life of 30 h. The hypothetical protein had an average GRAVY of 0.035, making the protein hydrophobic. The aliphatic index of the protein is 86.65, as the volume occupied by alanine, valine, leucine, and isoleucine is defined. Various physicochemical parameters of NHP1 are depicted in Table 1. Physical and chemical characterizations of the proteins used as positive and negative control were studied and are depicted in Tables S1 and S2.

**Table 1:** Physical and chemical characteristics of NHP1 protein predicted using EXPASY ProtParam server

Physicochemical parameters	Values
Number of amino acids	248
Molecular weight	26331.53 Da
pH	4.90
Theoretical isoelectric point (pI)	5.09
Formula	C_1167_H_1826_N_324_O_363_S_4_
Total number of atoms	3684
Aliphatic index	86.65
Instability index	44.06
Extinction coefficients (all pairs of Cys residues form cystines)	16055
Extinction coefficients (all Cys residues are reduced)	15930
Total number of negatively charged residues (Asp + Glu)	28
Total number of positively charged residues (Arg + Lys)	21
Grand average of hydropathicity (GRAVY)	0.035

### Conserved domain search

Conserved domains are sequences or structural units reoccurring in an evolutionary perspective. NHP1 nucleotide sequence subjected to NCBI BLAST showed 100% identity with many isolates of *M. tuberculosis* and 99% identity with many isolates of *M. canetti*. CDD-BLAST searches conserved domains by scanning the position-specific scoring matrices (PSSM) with all the conserved domains in the database with a protein query. CDD-BLAST, PFAM, and SMART did not show the presence of any conserved domain in the query protein.

### Subcellular localization

**Fig. 1:**
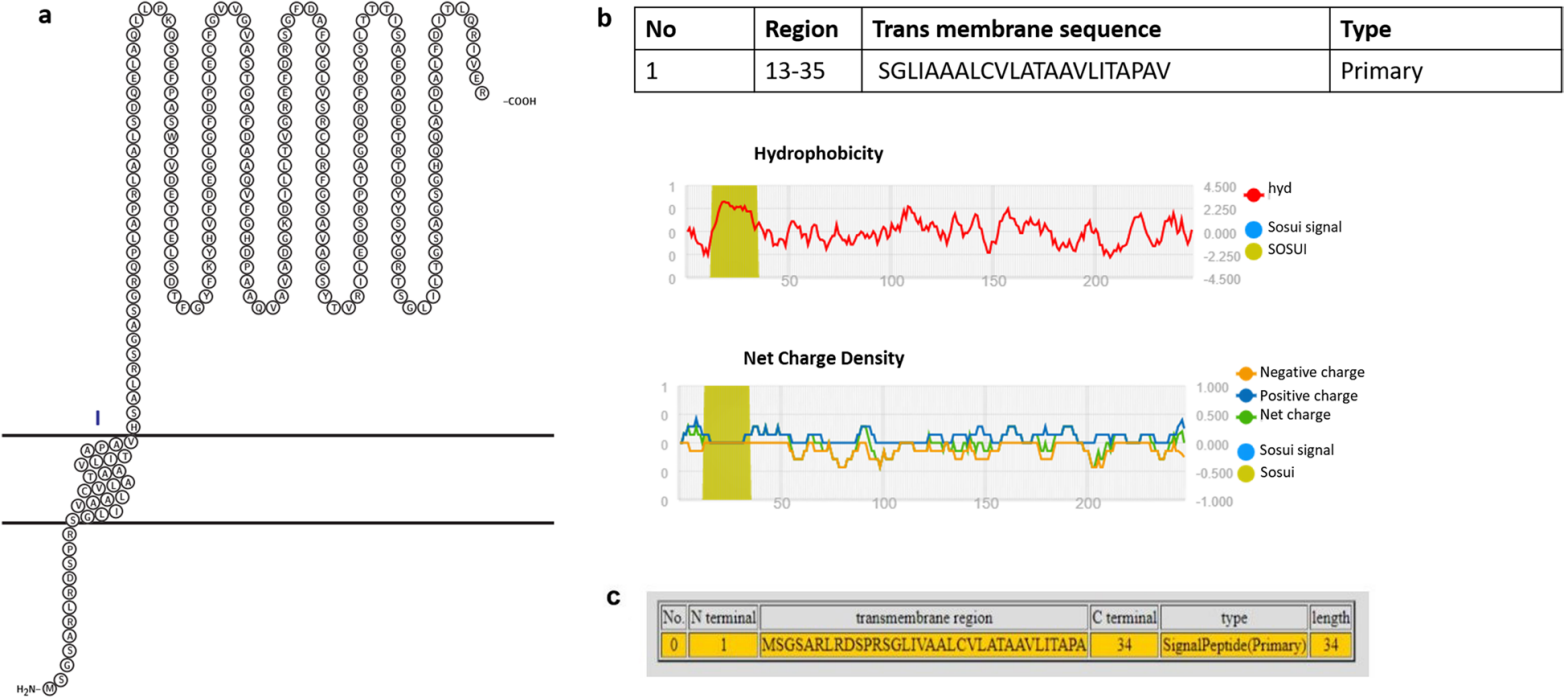
Cellular localization image of NHP1 protein showing a signal peptide with transmembrane structure using the server SOSUI. **a** N-terminal, transmembrane, and C-terminal domains of NHP1. **b** NHP1 hydropathy and charge plot. **c** Transmembrane sequence of NHP1

**Fig. 2:**
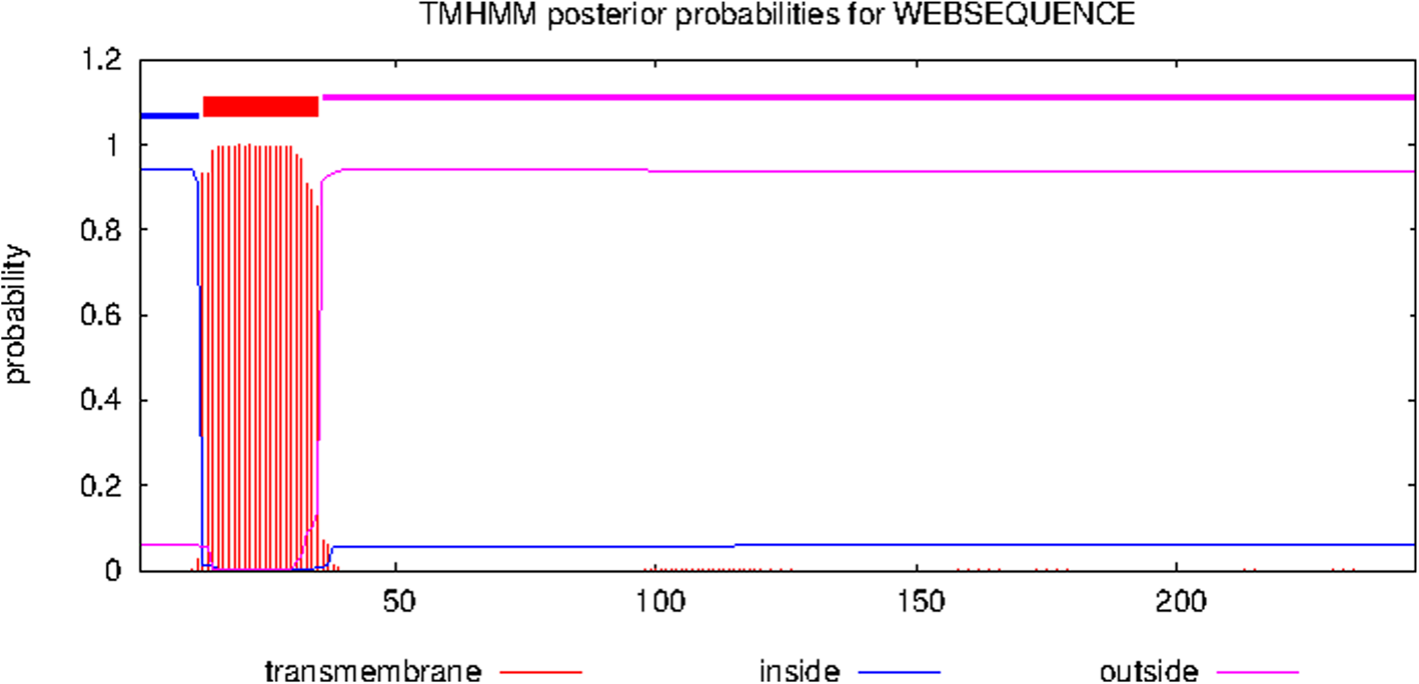
Graphical output of a transmembrane domain between 13 to 37 amino acids of NHP1 protein predicted using the server TMHMM

**Fig. 3:**
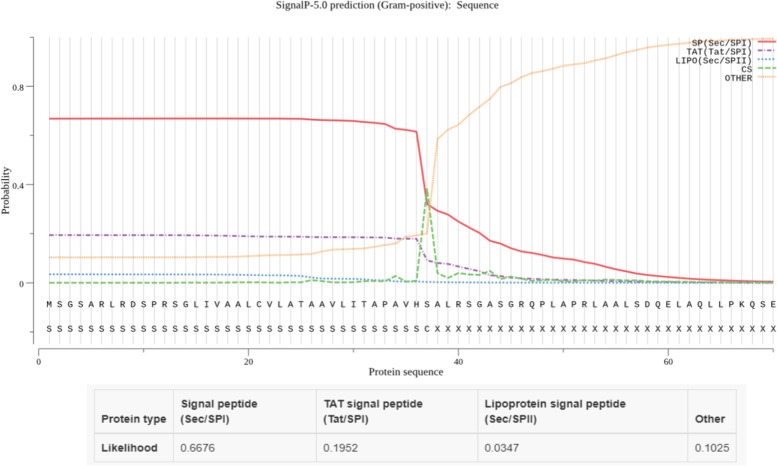
Graphical representation of the presence of signal peptide (sec/spl) with a cleave cite between 37 and 38 amino acids of the NHP1 protein using SignalP server

The 248 amino acid long sequence was used as the input in SOSUI, TMHMM, and CELLO to study the subcellular localization of the protein. These servers categorize the protein into transmembrane or cytoplasmic ones (Figs. 1 and 2). The results indicated the presence of signal peptide at the N-terminus and a transmembrane domain. Proteins used as positive and negative controls were studied using TMHMM server and did not show any transmembrane helices (Figs. S1 and S2). SignalP 5.0 and LipoP also predicted the presence of signal peptide and also a cleavage site between 37 and 38 amino acids of NHP1 (Fig. 3). SignalP 5.0 predicted the presence of signal peptide for esxA protein used as positive control but did not predict any signal peptide for other positive and negative control proteins (Figs. S3 and S4). InterPro tool predicted the presence of signal peptide and a transmembrane helix from regions 13–35 at N-terminus. The TBpred server also predicted the presence of an N-terminal signal peptide with transmembrane helices. PFP-FunDSeqE server predicted the query protein folding pattern to have flavodoxin-like folds, based on the functional domain and sequential evolutionary information. Evidence for ATP interacting residues were also found in the protein. The query NHP1 was thus predicted to be confined to the membrane and was found to possess a transmembrane domain in the N-terminal region with a signal peptide. Since the protein possesses these properties, immunological studies were further done.

### Prediction of antigenicity, allergenicity, toxicity, and virulence

VaxiJen servers predicted the protein as antigenic based on the physical and chemical properties, where the default threshold of 0.4 was chosen as the antigenicity measure. The query protein had an antigenicity score of 0.4509. The positive control proteins gave a VaxiJen threshold of esxA-0.557, esxB-0.782, and fbp-0.584 and negative control proteins whiB2-0.356, tuf ef-0.398, and cyp144-0.37, respectively. ANTIGENPro server predicted the antigenicity of the query protein as 0.764607. AllerTOP v.2.0 and AllergenFP predicted the query protein to be a probable non-allergen. Among the positive controls used, esxA protein was predicted as a probable allergen and toxic in nature, whereas esxB and fbpB proteins were nontoxic and non-allergen in nature. According to ToxinPred, whiB2 protein was studied as toxin, and tuf ef protein according to AllergenFP was studied as a probable allergen candidate. ToxinPred data predicted NHP1 protein to be nontoxic. VirulentPred data has shown NHP1 to be a virulent protein. Table 2 shows the comparison of immunological properties of NHP1 protein along with positive and negative controls.

**Table 2:** Comparison of the immunological properties of NHP1 protein with positive and negative controls

Prediction servers	NHP1	Positive control			Negative control		
		Rv3875	Rv3874	Rv1886c	Rv3260c	Rv0685	Rv1777
VaxiJen	0.450	0.557	0.782	0.584	0.356	0.398	0.370
AntigenPro	0.765	0.891	0.923	0.859	0.101	0.257	0.264
AllerTop	Non-allergen	Probable allergen	Non-allergen	Non-allergen	Non-allergen	Probable allergen	Non-allergen
AllergenFP	Non-allergen	Probable allergen	Non-allergen	Non-allergen	Non-allergen	Probable allergen	Non-allergen
ToxinPred	Nontoxic	Toxic	Nontoxic	Nontoxic	Toxic	Nontoxic	Non-toxic
VirulentPred	Virulent	Virulent	Virulent	Virulent	Virulent	Non-virulent	Virulent
HLAPred	Potential vaccine candidate	Potential vaccine candidate	Potential vaccine candidate	Potential vaccine candidate	Potential vaccine candidate	Can cause autoimmune disease	Potential vaccine candidate

### T-cell epitope prediction

HLAPred predicted 22 T-cell epitopes for the NHP1 protein. Predicted binders were scanned for genomic molecular mimicry against different species where no identical sequences were seen which can be used as a vaccine candidate. NetCTL server predicted the presence of seven MHC ligands with a threshold of 0.75 as shown in Table 3. IEDB MHC II binding predictions were used to predict the T-helper cell epitopes for allele HLA DR. The adjusted rank ranged from 0.02 to 100 where the recommended value is 2.2 where low adjusted rank ranging from 0.02 to 2.2 is considered good binders. All proteins chosen for positive and negative controls were studied for T-cell epitope binding property, and except fbpB protein, esxA and esxB proteins did not show the threshold value using NetCTL server and are shown in Table S3. T-cell epitope threshold values of all the negative controls using NetCTL are shown in Tables S4, S5, and S6. T-cell class I pMHC immunogenicity predictor depicted the immunogenicity of the protein to range from −0.02 to 1.09 threshold. T-cell class I pMHC immunogenicity threshold is shown in Table 4. pMHC class T-cell epitope studies of the positive and negative controls are given in Tables S7–S12.

**Table 3:** Sequence and threshold values of MHC ligands of NHP1 protein using NetCTL server

No	Sequence	Values
1	LSDQELAQL	0.8836
2	LSDTFGYFK	1.9021
3	FGSAVAGSY	0.8195
4	TAGPQRFRY	0.8411
5	PADETRTDY	1.6935
6	ETRTDYYSY	1.1664
7	RTDYYSYGR	0.7605

**Table 4:** Immunogenicity threshold value of NHP1 protein using T-cell class I pMHC immunogenicity predictor

Peptide	Length	Score
LAQLLPKQSEFPASWTVDETTELSDTFGYFKYHVFDEGLGFDPIECFGVVGVASTGAFDA	60	1.09703
AQVFGHDPAAQVAVADGKDILLTVGREFDRSGFDAFVGLVSRCLRFGSAVAGSYTVRILE	60	1.08736
DSRPTAGPQRFRYSLTTTISAEPADETRTDYYSYGRTSGLILTGSAGSGHQQALDALFDI	60	0.31786
TLQRIVER	8	0.26744
MSGSARLRDSPRSGLIAAALCVLATAAVLITAPAVHSALRSGASGRQPLAPRLAALSDQE	60	0.02806

### B-cell epitope prediction

**Fig. 4:**
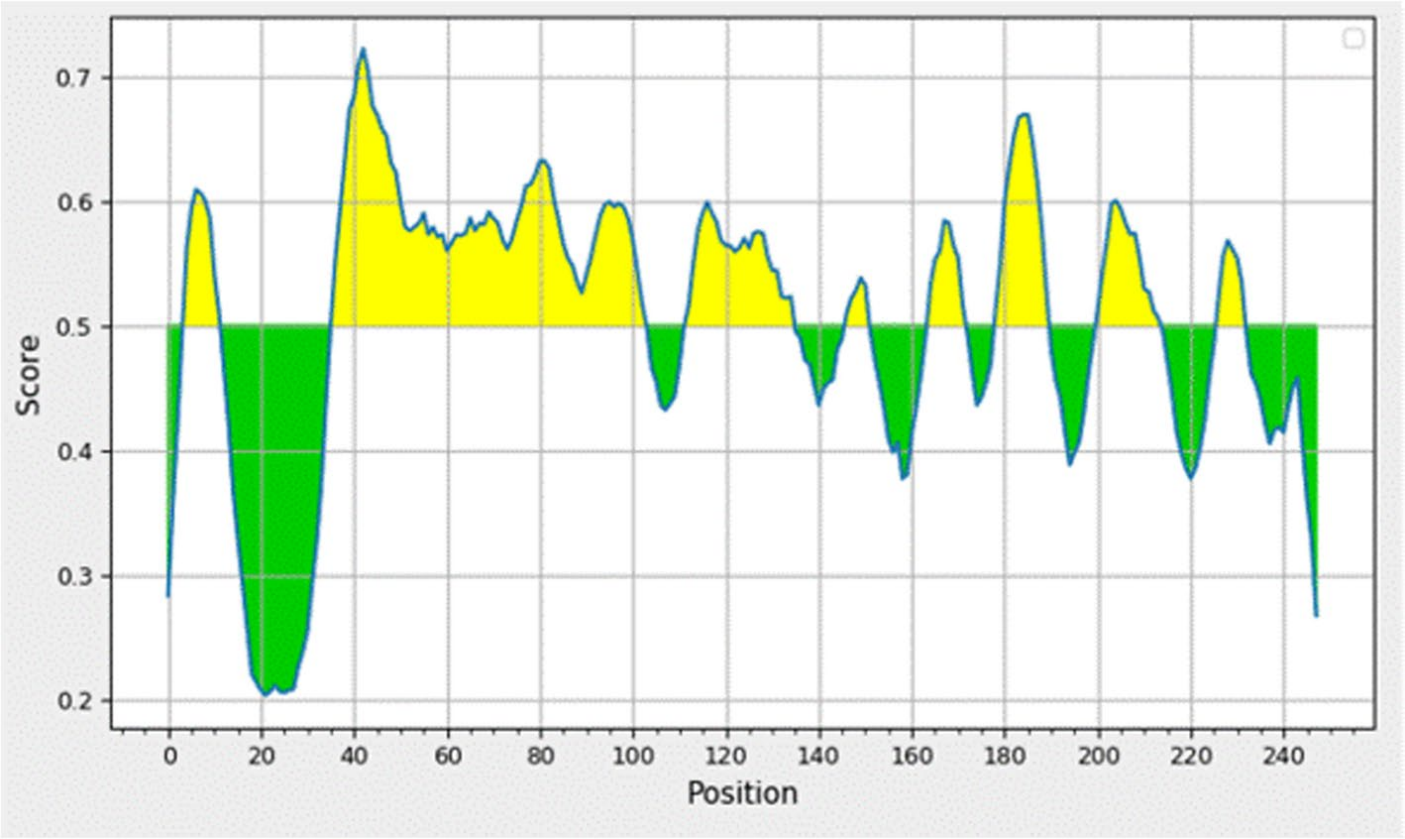
BepiPred threshold graph of NHP1 protein predicting linear epitopes of NHP1 protein. Yellow color depicts the predicted epitope residues

ABCpred was used to predict the B-cell epitope of the NHP1 protein using artificial neural networks. A threshold of 0.5 along with an epitope length of 16 amino acids was used in the ABCpred server. Eighteen epitopes were predicted by the ABCpred server above (0.92–0.56) the threshold level, where the default threshold value was 0.51. BcePred predicted the presence of six B-cell epitopes in the query protein with an accuracy of 58.7% at 2.3 threshold. Physicochemical characters like hydrophobicity, polarity, and flexibility were combined together at a threshold of 2.38 for linear B-cell epitope prediction by BcePred. BepiPred predicted the presence of B epitopes in the amino acid sequence of NHP1 with a threshold ranging from 0.2 to 0.722 (Fig. 4). BepiPred threshold of the positive and negative controls studied is depicted in Figs. S5 and S6.

### Protein secondary structure prediction

**Fig. 5:**
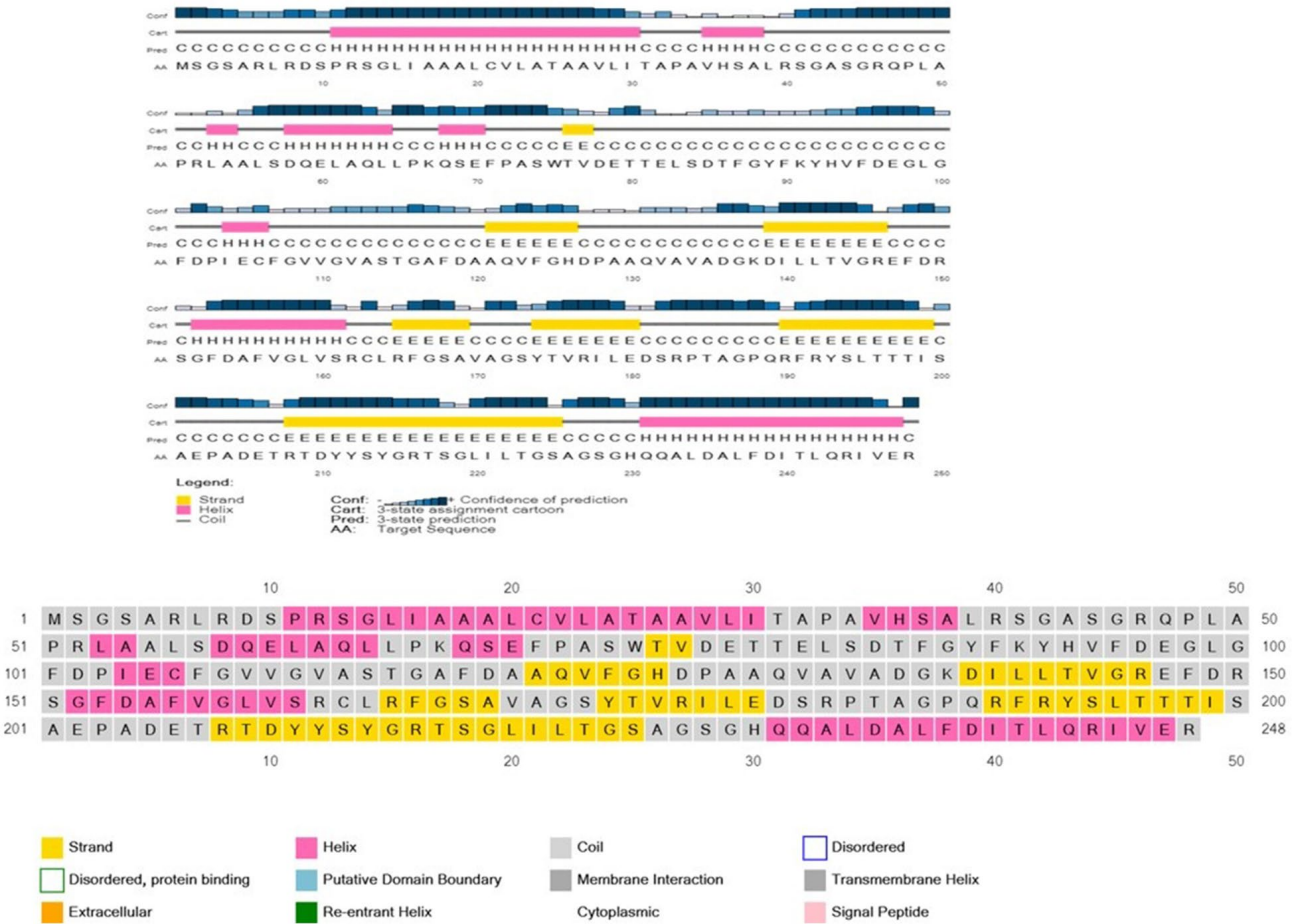
NHP1 protein secondary structure predicted by PSIPRED server displaying the presence of helix, strands, and random coils

The secondary structure of NHP1 was predicted using different computational tools (Table 5). SOPMA prediction is based on similar properties and evolutionary significance of homologous proteins in the databases with 69.5% prediction possibility. SOPMA predicted 33.47% alpha helical residues along with 18.55% extended strands, 3.23% beta turns, and 44.76% random coils. The GOR IV method is based on the probability of the amino acids to form a secondary structure. The GOR IV server predicted a similar structure as the SOPMA server. PSIPRED is an online secondary structure prediction tool which also predicts transmembrane topology, transmembrane helix, domain recognition sites, etc. PSIPRED also predicted strand, helix, and coiled secondary structure for the query protein (Fig. 5).

**Table 5:** Secondary structure elements developed by SOPMA and GRO4 server

Secondary structure elements	Symbol	No. of amino acids		Percentage	
		SOPMA	GORIV	SOPMA	GOR IV
Alpha helix	Hh	83	70	33.47%	28.23%
3_10_ helix	Gg	0	0	0.00%	0.00%
Pi helix	Ii	0	0	0.00%	0.00%
Beta bridge	Bb	0	0	0.00%	0.00%
Extended strand	Ee	46	55	18.55%	22.18%
Beta turn	Tt	8	0	3.23%	0.00%
Bend region	Ss	0	0	0.00%	0.00%
Random coil	Cc	111	123	44.76%	49.605

### Protein tertiary structure modeling

The conventional method of determining the three-dimensional protein structure is by NMR spectroscopy and X-ray diffraction. 3D homology models of NHP1 protein were predicted using the homology protein structure modeling method. Homology modeling has led to a new breakthrough in the field of computational biology. This modeling is very effective in studying molecular evolution since evolutionarily-related proteins share a common structure. MODELLER server is based on homology and comparative modeling of the protein sequence. MODELLER selected the most appropriate structure similar to NHP1 protein in the PDB database. The template 4TMD_A showed a probability of 99.56% with an E-value 1.8e-13 with a target length of 195. The predicted model was saved as PDB format. 3D structure of the protein was predicted using the Robetta server. Robetta is an automated tool for protein structure prediction using homology modeling and de novo structure prediction method.

**Fig. 6:**
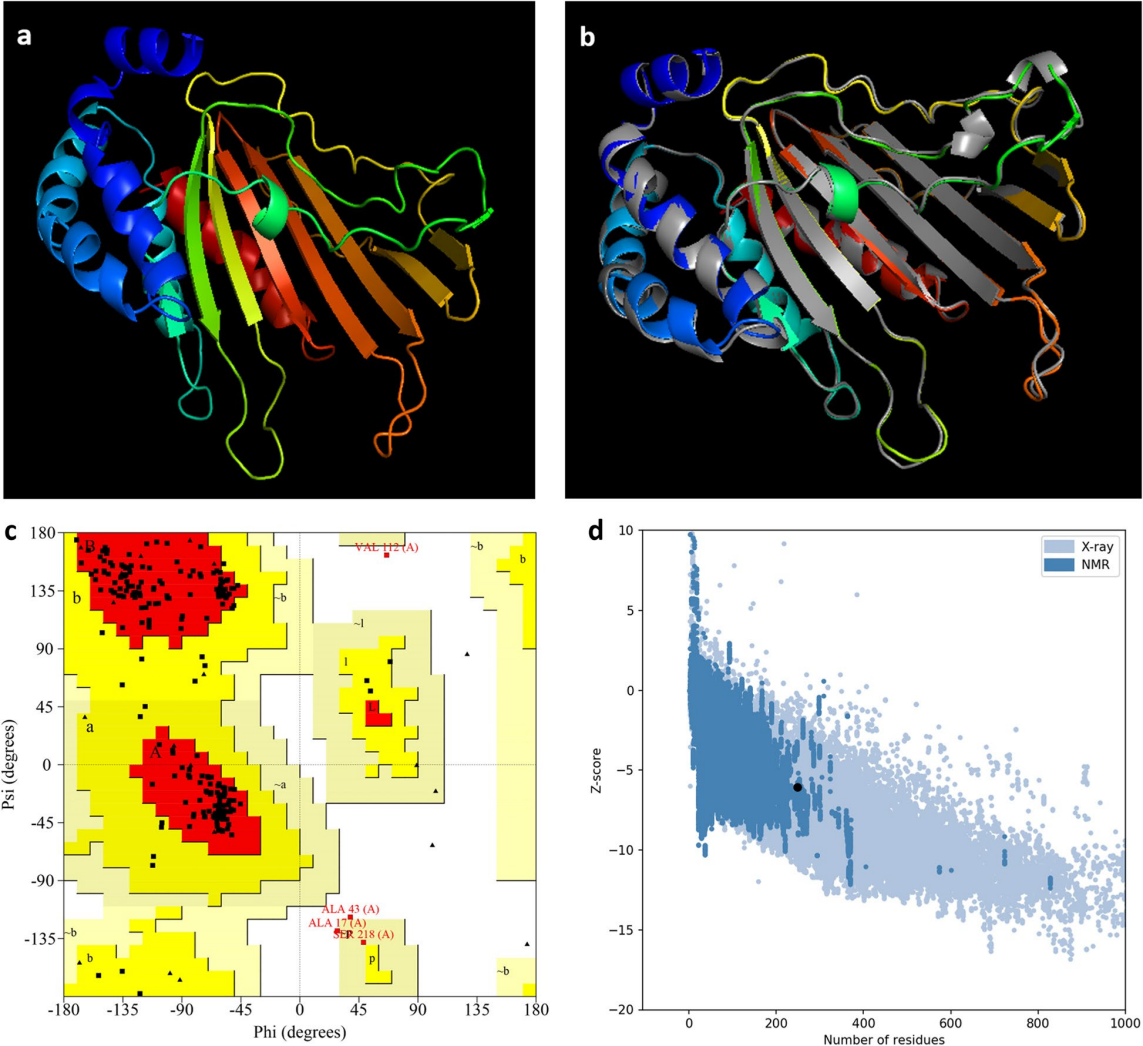
**a** The 3D model of NHP1 protein obtained from Robetta server following homology modeling. **b** Refined model after refinement superimposition using Galaxy Refine-GalaxyWEB server. **c** Ramachandran plot for structural validation with 92.7% residues in the favored region. **d** ProSA-web, with a Z score of −6.06

The predicted structures were analyzed using PROCHECK, VERIFY3D, and ProSA. Ramachandran plot was analyzed for the predicted model. The Ramachandran plot of the Robetta structure indicated 92.7% of residues with 188 residues in favorable regions and 20 residues in allowed regions. The number of non-glycine and non-proline residues was 212. ProSA web server was used for Z-score validation. The Z score of the 3-D sequence was estimated as −6.06. Analysis of the predicted structure using the VERIFY3D server showed that 80.24% of the residues have an average 3D-1D confirmation score. The refined image of the protein was predicted by the GalaxyWEB Refine server. GalaxyWEB Refine is used to improve the accuracy of the predicted model using side-chain perturbation. Refined 3D homology model of NHP1 had 95.1% residues as favored in the Ramachandran plot, with an RMSD value of 0.362 (Fig. 6).

## Discussion

Tuberculosis is one of the oldest infectious diseases which still causes extensive mortality and morbidity globally. The currently popular BCG vaccine is efficacious in imparting protection against childhood tuberculosis and TB meningitis, but is not very effective in preventing adult respiratory TB, and hence, there is an urgent need for identifying newer vaccine candidates. In silico studies of RD regions of *M. tuberculosis* depicted immunodominant properties. Genes encoded by RD1, RD7, and RD9 regions were found to be immunodominant antigens against T cells [48]. In the present in silico study, the hypothetical protein NHP1 mapped to be present in the RD7 region of *M. tuberculosis* was shown to encode a protein of molecular weight of 26.34 kDa, with highly acidic pH of 4.90 and an isoelectric point of 5.09. Physicochemical characterization of NHP1 protein showed a GRAVY score of 0.035 and an instability index of 44.06 making the protein hydrophobic. This also points to the fact that this could be a membrane protein of the bacteria. A high aliphatic index of 86.65 makes NHP1 thermostable as the greater the aliphatic index of a protein, the higher is its thermostability.

Three negative and three positive controls were used in the study for comparison of the physical, chemical, and immunological properties of the query protein. esxA (6 kDa early secreted antigen, encoded by Rv3875), esxB (protein activates human neutrophils, encoded by Rv3874), and fbpB (Ag85B elicits cellular immunity, encoded by Rv1886c) which were extensively studied for immunological properties both in silico and in vivo and further subjected to vaccine studies were selected as positive control. whiB2 (Rv3260c), tuf elongation factor (Rv0686), and cyp144 (Rv1777) proteins that are important for the biochemical functions of the bacteria were used as negative control.

Subcellular localization studies play an important role in identifying proteins as drug targets, and most of the prediction tools are validated against mycobacterial proteins [49]. NHP1 protein was predicted to contain a signal sequence with transmembrane helices attached to the membrane. Previous studies conducted on twelve P450 enzymes of *M. tuberculosis* using different computational tools like CELLO, TMHMM, and HMMTOP predicted these proteins as cytoplasmic transmembrane proteins with no signal peptides. Homology modeling of the proteins was done using RaptorX, SWISS-MODEL server, and Phyre 2. Molecular docking of the proteins was done with azole drugs using AutoDock Vina and revealed that the P450 enzymes were found to act as a target site for novel drugs. Different azole drugs docked against P450 enzymes were predicted to be good drug candidates against *M. tuberculosis* [50]. According to the PSIPRED tool, NHP1 had an extracellular C-terminal region, a transmembrane helix, and an N-terminal region attached to the membrane. *M. tuberculosis* membrane proteins are known to be immunogenic. Surface membrane proteins are considered as potential vaccine targets, while cytoplasmic proteins are possible drug targets [51]. Since membrane-associated proteins of *M. tuberculosis* are generally potent activators of human T-cell responses [3], NHP1 also could be thought of as a potential vaccine candidate.

A study of 999 hypothetical proteins of *M. tuberculosis* H37Rv was published in 2016 which can be used for comparative studies. These 999 hypothetical proteins were further studied for conserved domains using CDD Blast and ScanProsite. Ninety-eight proteins were selected from this set and further studied for structural and functional characterization. Proteins with plasma membrane localization and transmembrane topology prediction were studied for antigenicity using VaxiJen server. Proteins having an antigenic score of > 5 were considered and selected for epitope prediction of which two proteins were shown to be having 15 linear epitopes and 21 conformational epitopes. This study has shown these epitopes can be candidates for developing new drugs or vaccines against tuberculosis [15].

ATPint: tool predicted the ATP binding residues of the NHP1 protein (https://webs.iiitd.edu.in/cgibin/atpint/chkres?8477). PFP-FunDSeqE server results depict NHP1 protein to have a flavodoxin-like folding. Proteins with flavodoxin-like folding patterns are considered as ancient origin structures. Flavodoxin family proteins of *M. bovis* BCG, *M. canetti*, *M. pinnipedii*, and few other species of *Mycobacterium* were found to be deposited in EMBL UniProt (https://www.uniprot.org/uniref/UniRef100_ A0A0K2HZ71). Flavin cofactors were present in flavodoxin folding proteins in the electron transport chain, and in few bacteria, these proteins were induced in low iron conditions [52].

Antigenicity refers to the ability to recognize a specific antigen followed by an immune response, whereas immunogenicity is the ability to induce cellular and humoral immune response. An ideal vaccine candidate should be both antigenic and immunogenic [53]. Immunological properties of NHP1 protein were studied, predicting the protein to be highly antigenic, having epitopes to both T-cell and B-cell receptors. T-helper cells play a major role in providing immunity against *M. tuberculosis*. T-cell epitope prediction of proteins including ESAT-6, CFP10, Ag85B, and MPT70 was studied using ProPred server [54]. PPE65 family proteins were studied using immunoinformatics and were predicted to be vaccine candidate proteins [55]. For VaxiJen, all the positive control proteins should possess an antigenic score of > 0.4 and can be subjected to vaccine studies, but the negative control proteins should have an antigenic score of < 0.4 making them nonantigenic. The antigenic threshold of NHP1 protein was 0.45, and hence when compared to the positive and negative controls, the query protein can be used as a vaccine candidate. The antigenic threshold of the positive controls was as follows: esxB (0.782), fbpB (0.584), and esxA (0.557), and negative controls were whiB2 (0.35), tuf elongation factor (0.398), and cyp144 (0.37). The antigenic threshold of NHP1 found using the server Antigen Pro was 0.765.

Allergenicity studies provide clues regarding the possible allergic reactions stimulated by the vaccine. AllerTOP data has shown that NHP1 is nonallergenic. According to AllerTOP and AllergenFP, the positive control proteins esxB and CFP10 protein were nonallergenic and nontoxic, whereas esxA protein was a probable allergen and toxic in nature. In the present study, NHP1 was predicted to have high binding affinity towards B and T cells, showed allergenicity and antigenicity, and was also nonallergenic. Hence, the protein could be considered as a potential vaccine candidate. B-cell epitopes generally play an important role in eliciting humoral immunity and hence are significant in vaccine design too. All positive control proteins showed the presence of more epitopes binding to both T and B cells. According to NetCTL server, NHP1 protein showed 7 MHC ligands above the threshold value, whereas fdpB (positive control) showed 6 MHC ligands. Only 2 MHC ligands above the threshold value were found in all three negative controls under study. IEDB MHC II predicted the presence of T-helper cell epitopes of which fbpB and cyp144 were having T-helper cell epitopes. BepiPred predicted the presence of B epitope alleles for positive and negative controls. In comparison with all positive and negative controls, NHP1 protein showed immunological properties which tend to make it a vaccine candidate.

The secondary structure of NHP1 was predicted using SOPMA, PSIPRED, and GOR-IV. SOPMA and PSIPRED were earlier used to predict the structure of the hypothetical protein CGM946K2_146 of *M. tuberculosis*. The 3D structure of NCGM946K2_146 gene of *M. tuberculosis* was studied using tools like SWISS-MODEL server, Phyre2, and MODELLER, of which MODELLER was found to be the most efficient tool for homology protein structure prediction. The structural and functional studies of NCGM946K2_146 pointed to those of a potential therapeutic drug candidate [56]. PSIPRED was used to study the secondary structure of Rv1907c gene of *M. tuberculosis H37Rv* [57]. Structural studies of alpha-phosphoglucomutase of *M. tuberculosis* were carried out using GOR-IV server. SOPMA and GOR IV predicted 28–33% of alpha helical structure, 18–22% extended strand structure and 44–49% random coil structure, and 3% beta turn structure. PSIPRED predicted the secondary structure similar to the prediction of GOR-IV and SOPMA with high confidence of prediction. The tertiary structure of the NHP1 protein was studied using homology modeling. Membrane proteins including NP_216679, NP_218309, and NP_218312 of *M. tuberculosis* were studied for physical and chemical characterization, secondary structure prediction, and functional characterization using different computer tools. These proteins were found to be membrane proteins with transmembrane helices.

3D structural studies of NHP1 were done using homology protein modeling. Proteins with high sequence similarity were selected as templates using the MODELLER server. Along with the homology template model, a position-specific scoring matrix (PSSM) and hidden Markov model (HMM)-based server Robetta were considered to develop the 3D structure of the query protein. The developed structures were refined and validated using different quality assessment tools including PROCHECK. The refinement of the developed 3D structure of the protein was also done using the Galaxy web server which is based on molecular dynamic simulation.

The physical and chemical parameters of a multiepitope vaccine developed using reverse vaccine technology were studied using Expasy ProtParam which predicted the vaccine developed as acidic, hydrophilic, and stable in nature [16]. Another study using computational tools had revealed that NCGM946K2_146 protein with 455 amino acids was acidic and unstable in nature as per the physical and chemical parameters [56]. 4TMD_A (PDB ID), a hypothetical protein of *M. smegmatis*, showed 99.56% sequence similarity with 195 amino acids among the 248 long amino acid NHP1 sequence. Robetta server was used to predict the structure of NHP1 using 4TMD_A as model protein structure. 3D structure of a multiepitope vaccine using Ag85A (Rv3804c), Mtb32A (Rv0125), Rv2684, and Rv2608 developed previously using ITASSER showed 85.9% favored region, 8.9% allowed region, and 5.2% disallowed region in Ramachandran plot [16]. Ramachandran plot for NHP1 protein structure favored 84% of the peptide residues in the favorable region, and 8% of the residue in the allowed regions confirming the phi and psi bond angles and secondary structure prediction was complementary. The VERIFY3D server was used to compare the one-dimensional and three-dimensional structure of the predicted protein, giving a result of 80.02%. GalaxyWEB Refine server was used to refine the predicted model structure, allowing a 3% refinement further showing 95.1% residues in the Ramachandran plot to be favorable. If at least 90% of its residues lie in the favored region, the protein model can be considered reliable [58]. Since the total residues in the favored region of NHP1 model were more than 90%, it can be considered a valid structure. In another study of modeled structures, NP_218312 was docked against different ligands in search of potential drug candidates against tuberculosis [59].

Homology protein modeling has led to a new breakthrough in the field of computational biology. In homology modeling, new protein structures are designed using a known protein structure. This method is very effective in studying evolutionarily related proteins since they share common structural domains. Crystallographic studies of membrane proteins are challenging, since they can be affected by detergents and other physical and chemical properties of the protein. Total number of protein structures deposited in Protein Data Bank (PDB) until 2021 is 183,584 of which 2037 are membrane proteins. Generally, a sequence identity more than 50% can give reliable models with only slight errors in the loops and side-chain positions. Homology protein modeling is a time-saving method when compared to X-ray crystallography technique.

Cell-mediated response is significant in the progress of the disease in a patient. Bacterial membrane proteins play a significant role in the host-pathogen interaction. Hence, the protein under study offers a promise of immunogenicity and hence important as a candidate vaccine. The 3D structure of NHP1 was predicted with 99% confidence using the Robetta server. NHP1 protein has also shown 99% sequence similarity with *M. canetti*, which is an ancestral smooth tubercle bacillus*.* Studies on the nature of protein folds have given us insights on the presence of a flavodoxin-like fold in NHP1 pointing to the ancient nature of this mycobacterial protein, a corroboration of our earlier research on the N4.5 region’s close similarity with the corresponding genomic locus in the smooth tubercle bacillus, *M. canettii*. Thus, the evidence collected through various in silico approaches to better understand NHP1 has led us to the conclusion that it is a transmembrane, signal peptide of *M. tuberculosis* with sufficient antigenicity and immunogenicity as depicted by prediction servers like VaxiJen, AntigenPro, AllerTOP, and HLAPred. An ideal vaccine to put a curb on the spread of tuberculosis should target multiple immune system components. As it is very difficult to create a vaccine with all these attributes, multiple vaccines or combination approaches would be the ideal strategy to look forward to. In this context, the current study exploring the possibility of NHP1 as a potential vaccine candidate assumes importance as it has immunogenic features comparable with those of esxA, esxB, Ag85B, etc. which are currently explored vaccine candidates.

## Conclusion

The structural and functional properties of a putative protein, NHP1, in the RD7 region of the genome were studied using in silico approaches. This was found to be an ORF in a subtracted genomic region N4.5 in clinical isolates in India. The initial studies on this protein depicted it to be a novel multiepitope possible subunit vaccine candidate. NHP1, predicted to be a membrane protein with signal sequence, showed 99% similarity with the counterpart in *M. canetti* and showed the presence of flavodoxin-like folding patterns with ATP binding sites stressing the evolutionarily ancient nature of the protein. Evidence from our earlier studies of the sequences upstream and downstream of NHP1 loci has also revealed almost 100% similarity with the corresponding regions of *M. canetti*, the ancient smooth tubercle bacillus. These two facts drive home the ancient nature of this locus. This revelation is particularly important as India has a lot of clinical isolates of the pathogen falling under the category of ancestral strains. NHP1 protein showed high antigenic threshold along with epitopes of T cells and B cells making the protein a vaccine candidate similar to esxB and fbpB proteins and other proteins in the RD7 region. The comparative analysis of NHP1 protein with positive and negative controls showed that NHP1 protein has comparable immunological properties and hence is likely to have vaccine potential. Data from these computational studies have revealed NHP1 to be a membrane protein with good antigenicity and non-allergenicity. Thus, the current approach has helped in identifying a multiepitope protein present in the RD7 region of *M. tuberculosis* clinical strains as a novel vaccine candidate using immunoinformatics tools which can be further analyzed using animal models for its efficacy.

## Supplementary Information

**Additional file 1.**

## Data Availability

All the data and material generated and analyzed in this study have been included in this manuscript

## Abbreviations

BCG: Bacille Calmette-Guérin
ESAT-6: Early secretory antigenic target-6
RD: Region of difference
HIV: Human immunodeficiency virus
TB: Tuberculosis
mce3: Mammalian cell entry
SMRT: Single-molecule, real-time
MTBC: *Mycobacterium tuberculosis* complex
GTP: Guanosine triphosphate
PCR: Polymerase chain reaction
NCBI: National Center for Biotechnology Information
BLAST: Basic local alignment search tool
ORF: Open reading frame
SMS: Sequence manipulation suite
CDD-BLAST: Conserved domain database
SMART: Simple modular architecture research tool
TMHMM: Tied mixture hidden Markov model
SOSUI: Software systems for prediction of signal peptide and membrane protein
CELLO: SubCellular localization predictor
HLA Pred: Human leukocyte antigen
MHC-I: Major histocompatibility complex-I
HTL: Helper T lymphocytes
pMHC: Peptide-bound MHC
IEDB: Immune epitope database
HMM: Hidden Markov model
PSIPRED: PSI-blast-based secondary structure prediction
SOPMA: Self-optimized prediction method with alignment
GOR IV: Garnier–Osguthorpe–Robson 4
PDB: Protein Data Bank
ProSA: Protein structure analysis
CYS: Cysteine
Asp: Aspartic acid
Glu: Glutamic acid
Arg: Arginine
Lys: Lysine
GRAVY: Grand average of hydropathicity
