# Supplementary material for: Structural and functional characterization of a hypothetical protein in the RD7 region in clinical isolates of Mycobacterium tuberculosis — an in silico approach to candidate vaccines
Source: J Genet Eng Biotechnol. 2022 Apr 8;20:55. doi: 10.1186/s43141-022-00340-5 (PMC8993957; doi:10.1186/s43141-022-00340-5)

**Supplementary data**

Table S1. Physical and chemical characteristics of positive controls predicted using EXPASY Protparam server

| **Physico-chemical parameters** | esxB | fbpB | esxA |
| --- | --- | --- | --- |
| Number of amino acids | 100 | 325 | 288 |
| Molecular weight | 10793.80 Da | 34580.85 | 23038.08 |
| pH | 4.39 | 4.50 | 5.14 |
| Theoretical isoelectric point (pI) | 4.59 | 5.62 | 5.26 |
| Chemical formula | C_454_H_730_N_138_O_161_S_3_ | C_1547_H_2334_N_418_O_461_S_13_ | C_812_H_1338_N_288_O_333_S80 |
| Total number of atoms | 1486 | 4773 | 2851 |
| Aliphatic index | 63.70 | 72.18 | 23.61 |
| Instability index | 41.90 | 44.93 | 44.73 |
| Extinction coefficients (all pairs of Cys residues form cystines) |  |  |  |
| Extinction coefficients (all Cys residues are reduced) | 6990 | 81360 | 5000 |
| Total number of negatively charged residues (Asp + Glu) | 14 | 23 | 0 |
| Total number of positively charged residues (Arg + Lys) | 9 | 20 | 0 |
| Grand average of hydropathicity (GRAVY) | -0.668 | -0.176 | 0.879 |

Table S2. Physical and chemical characteristics of negative controls predicted using EXPASY Protparam server

| **Physico-chemical parameters** | whiB2 | tuf | cyp144 |
| --- | --- | --- | --- |
| Number of amino acids | 89 | 396 | 434 |
| Molecular weight | 10138.48 Da | 43561.50 | 47186.84 |
| pH | 10.42 | 4.70 | 4.84 |
| Theoretical isoelectric point (pI) | 5.19 | 5.28 | 5.17 |
| Formula | C_445_H_693_N_129_O_133_S_5_ | C_1920_H_3088_N_540_O_594_S_10_ | C_2090_H_3303_N_593_O_619_S_17_ |
| Total number of atoms | 1405 | 6153 | 6622 |
| Aliphatic index | 64.83 | 89.52 | 94.54 |
| Instability index | 41.90 | 28.30 | 34.89 |
| Extinction coefficients (all pairs of Cys residues form cystines) | 12740 |  | 50670 |
| Extinction coefficients (all Cys residues are reduced) | 12490 | 15930 | 50420 |
| Total number of negatively charged residues (Asp + Glu) | 17 | 60 | 56 |
| Total number of positively charged residues (Arg + Lys) | 13 | 45 | 40 |
| Grand average of hydropathicity (GRAVY) | -0.703 | -0.283 | -0.018 |

Figure S1: Graphical output of a transmembrane domain of amino acids of esx A, esx B and fbpB protein used as positive controls predicted using the server TMHMM


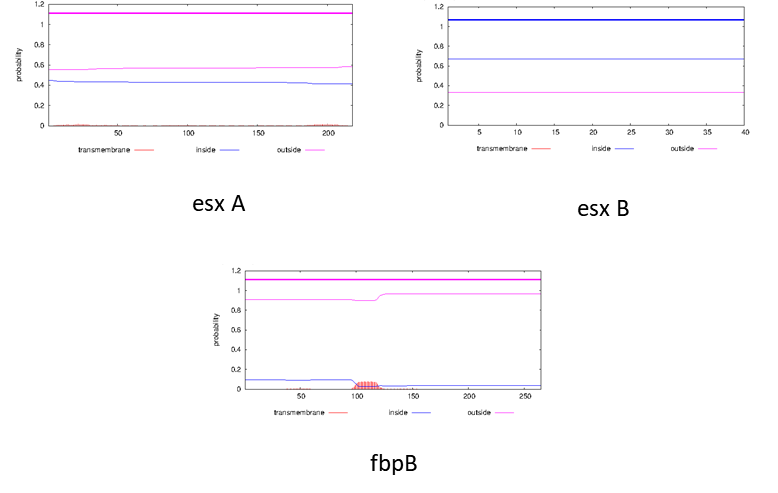


Figure S2: Graphical representation of a transmembrane domain of amino acids of the whiB2, tuf ef and cyp114 proteins used as negative controls predicted using the server TMHMM


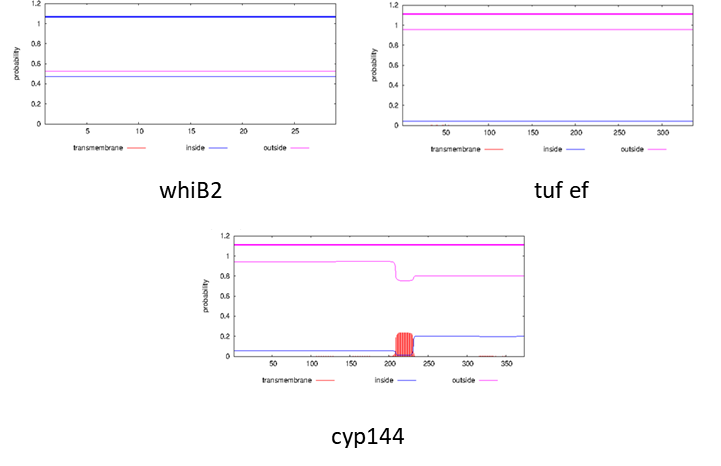


Figure S3: Graphical representation of the presence of signal peptide (sec/spl) of esxA, esxB and fbpB Proteins used as positive control developed by Signal P server.


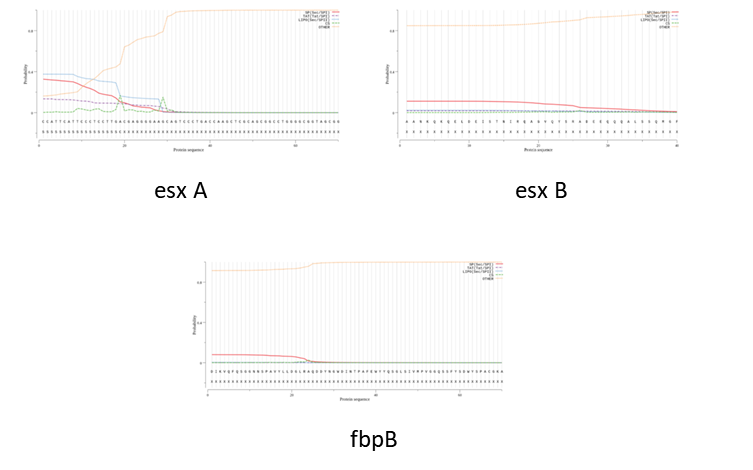


Figure S4: Graphical representation of the presence of signal peptide (sec/spl) of whiB2, tuf ef and cyp144 Proteins used as negative control developed by Signal P server.


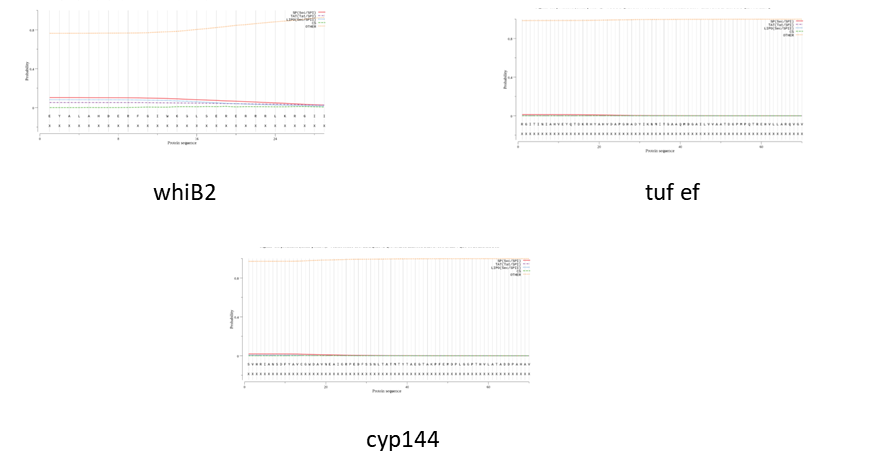


Table S3: Sequence and Threshold values of MHC ligands of fbpB protein used as positive control predicted using NetCTL server.

| **No** | **Sequence** | **Values** |
| --- | --- | --- |
| 1 | NTPAFEWYY | 2.8735 |
| 2 | PVGGQSSFY | 0.9016 |
| 3 | QSSFYSDWY | 3.0360 |
| 4 | YSDWYSPAC | 0.8301 |
| 5 | SSAMILAAY | 2.5007 |
| 6 | LAMGDAGGY | 0.9212 |
| 7 | ANNTRLWVY | 0.7677 |

Table S4: Sequence and Threshold values of MHC ligands of whiB2 protein used as a negative control predicted using NetCTL server.

| **No** | **Sequence** | **Values** |
| --- | --- | --- |
| 1 | ATDQWQDRA | 1.0735 |
| 2 | QTDPEAFFP | 0.9222 |

Table S5: Sequence and Threshold values of MHC ligands of tuf ef protein used as a negative control predicted using NetCTL server.

| **No** | **Sequence** | **Values** |
| --- | --- | --- |
| 1 | TINIAHVEY | 1.4859 |
| 2 | QTDKRHYAN | 1.6472 |
| 3 | QMDGAILVV | 1.0171 |
| 4 | TTVTGVEMF | 0.7839 |
| 5 | GTTTPHTEF | 0.7776 |
| 6 | HTEFEGQVY | 3.3431 |
| 7 | TTDVTGVVT | 0.8438 |

Table S6: Sequence and Threshold values of MHC ligands of cyp144 protein used as a negative control predicted using NetCTL server

| **No** | **Sequence** | **Values** |
| --- | --- | --- |
| 1 | GAESVQDPY | 1.2688 |
| 2 | SVQDPYPLY | 1.0442 |
| 3 | FSSNLTATM | 0.8577 |
| 4 | IAQLVKWGY | 1.0195 |
| 5 | LMELSGYIF | 0.7861 |
| 6 | ATACASGEL | 0.8153 |

Table S7: Immunogenicity threshold value of esxB protein used as a positive control using T cell class I pMHC immunogenicity predictor

| **Peptide** | **Length** | **Score** |
| --- | --- | --- |
| MAEMKTDAATLAQEAGNFERISGDLKTQIDQVESTAGSLQGQWRGAAGTAAQAAVVRFQE | 60 | 0.29054 |
| AANKQKQELDEISTNIRQAGVQYSRADEEQQQALSSQMGF | 40 | -1.11354 |

Table S8: Immunogenicity threshold value of fbpB protein used as a positive control using T cell class I pMHC immunogenicity predictor

| **Peptide** | **Length** | **Score** |
| --- | --- | --- |
| VANNTRLWVYCGNGTPNELGGANIPAEFLENFVRSSNLKFQDAYNAAGGHNAVFNFPPNG | 60 | 1.13241 |
| MTDVSRKIRAWGRRLMIGTAAAVVLPGLVGLAGGAATAGAFSRPGLPVEYLQVPSPSMGR | 60 | 0.48701 |
| DIKVQFQSGGNNSPAVYLLDGLRAQDDYNGWDINTPAFEWYYQSGLSIVMPVGGQSSFYS | 60 | -0.08665 |
| THSWEYWGAQLNAMKGDLQSSLGAG | 25 | -0.27309 |
| QQFIYAGSLSALLDPSQGMGPSLIGLAMGDAGGYKAADMWGPSSDPAWERNDPTQQIPKL | 60 | -0.60226 |
| DWYSPACGKAGCQTYKWETFLTSELPQWLSANRAVKPTGSAAIGLSMAGSSAMILAAYHP | 60 | -0.73126 |

Table S9: Immunogenicity threshold value of esxA protein used as a positive control using T cell class I pMHC immunogenicity predictor

| **Peptide** | **Length** | **Score** |
| --- | --- | --- |
| AACCTGGCGCGGACGATCAGCGAAGCCGGTCAGGCAATGGCTTCGACCGAAGGCAACGTCACTGGGATGT | 70 | 0.71994 |
| ATGACAGAGCAGCAGTGGAATTTCGCGGGTATCGAGGCCGCGGCAAGCGCAATCCAGGGAAATGTCACGT | 70 | 0.9498 |
| CCATTCATTCCCTCCTTGACGAGGGGAAGCAGTCCCTGACCAAGCTCGCAGCGGCCTGGGGCGGTAGCGG | 70 | 0.50286 |
| TCGCATAG | 8 | 0.06441 |
| TTCGGAGGCGTACCAGGGTGTCCAGCAAAAATGGGACGCCACGGCTACCGAGCTGAACAACGCGCTGCAG | 70 | 0.65316 |

Table S10: Immunogenicity threshold value of whiB2 protein used as a negative control using T cell class I pMHC immunogenicity predictor

| **Peptide** | **Length** | **Score** |
| --- | --- | --- |
| LVPEAPAPFEEPLPPEATDQWQDRALCAQTDPEAFFPEKGGSTREAKKICMGCEVRHECL | 60 | 0.7305 |
| EYALAHDERFGIWGGLSERERRRLKRGII | 29 | 0.95792 |

Table S11: Immunogenicity threshold value of tuf ef protein used as a negative control using T cell class I pMHC immunogenicity predictor

| **Peptide** | **Length** | **Score** |
| --- | --- | --- |
| EGDAKWVASVEELMNAVDESIPDPVRETDKPFLMPVEDVFTITGRGTVVTGRVERGVINV | 60 | 1.37357 |
| TTPHTEFEGQVYILSKDEGGRHTPFFNNYRPQFYFRTTDVTGVVTLPEGTEMVMPGDNTN | 60 | 1.11675 |
| HVLLARQVGVPYILVALNKADAVDDEELLELVEMEVRELLAAQEFDEDAPVVRVSALKAL | 60 | 0.92326 |
| ISVKLIQPVAMDEGLRFAIREGGRTVGAGRVTKIIK | 36 | 0.73418 |
| RGITINIAHVEYQTDKRHYAHVDAPGHADYIKNMITGAAQMDGAILVVAATDGPMPQTRE | 60 | 0.59002 |
| VAKAKFQRTKPHVNIGTIGHVDHGKTTLTAAITKVLHDKFPDLNETKAFDQIDNAPEERQ | 60 | 0.48796 |
| NEEVEIVGIRPSTTKTTVTGVEMFRKLLDQGQAGDNVGLLLRGVKREDVERGQVVTKPGT | 60 | 0.40738 |

Table S12: Immunogenicity threshold value of cyp144 protein used as a negative control using T cell class I pMHC immunogenicity predictor

| **Peptide** | **Length** | **Score** |
| --- | --- | --- |
| RANPELLGAFIEETLRYEPPFRGHYRHVRNATTLDGTELPADSHLLLLWGAANRDPAQFE | 60 | 1.65891 |
| APGEFRLDRAGGKGHISFGKGAHFCVGAALARLEARIVLRLLLDRTSVIEAADVGGWLPS | 60 | 1.55752 |
| MVVAELIGLPDPDIAQLVKWGYAATQLLEGLVENDQLVAAGVALMELSGYIFEQFDRAAA | 60 | 1.03037 |
| SVHRIANSDFYAVCGWDAVNEAIGRPEDFSSNLTATMTYTAEGTAKPFEMDPLGGPTHVL | 60 | 1.01664 |
| ATADDPAHAVHRKLVLRHLAAKRIRVMEQFTVQAADRLWVDGMQDGCIEWMGAMANRLPM | 60 | 0.5878 |
| DPRDNLLGELATACASGELDTLTAQVMMVTLFAAGGESTAALLGSAVWILATRPDIQQQV | 60 | 0.52814 |
| ILVRRIERLELAVQ | 14 | 0.52678 |
| VRRSPKGSPGAVLDLQRRVDQAVSADHAELMTIAKDANTFFGAESVQDPYPLYERMRAAG | 60 | -0.18577 |

Figure S5: BepiPred threshold graph of esxA, esxB and fbpB proteins used as positive controls predicting linear epitopes. Yellow color depicts the predicted epitope residues.


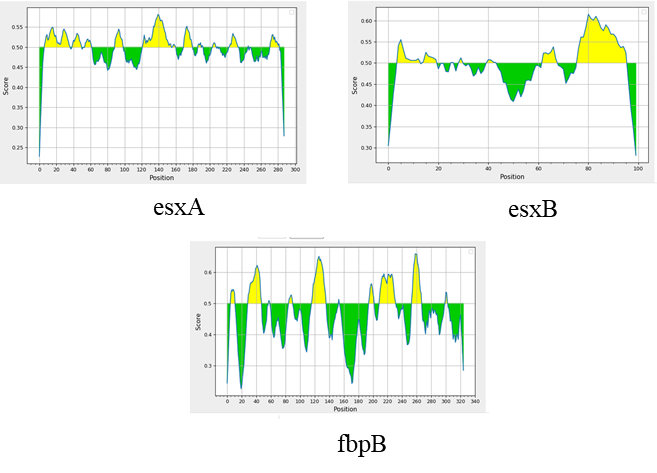


Figure S6: BepiPred threshold graph of whiB2, tuf ef and cyp144 proteins used as negative controls predicting linear epitopes. Yellow color shows the predicted epitope residues with threshold value.


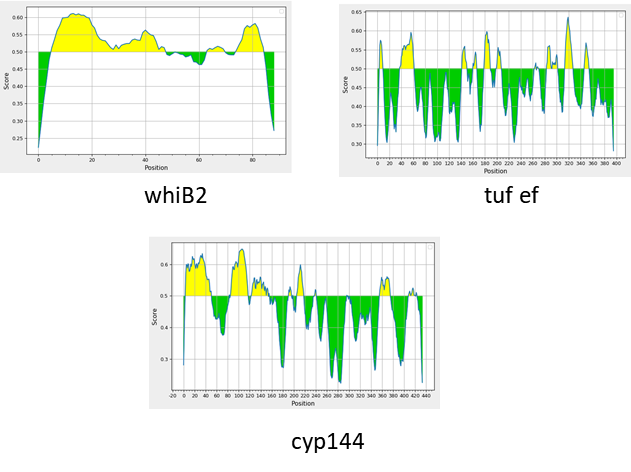

Supplement: Supplementary file 1 — Additional file 1. [file 43141_2022_340_MOESM1_ESM.docx]
